# Balancing act: Studying the effect of perch space allowance on welfare in Canadian laying strain pullets raised in floor pens with access to a single-tier perch system to 18 wk of age

**DOI:** 10.1016/j.psj.2024.104457

**Published:** 2024-10-30

**Authors:** Carolin A.B. Adler, Tory Shynkaruk, Samantha McPhee, Kailyn Buchynski, Adelle Herr, Eugenia Herwig, Karen Schwean-Lardner

**Affiliations:** Department of Animal and Poultry Science, University of Saskatchewan, Saskatoon, Saskatchewan, Canada, S7N 5A8

**Keywords:** Behavior, Health, Performance, Perch usage, Body width

## Abstract

The rearing environment for pullets should mirror their later production environment as closely as possible. However, existing perch space recommendations are based on data for laying hens rather than pullets. This study explores the impact of perch space allowances on the welfare of Canadian laying strain pullets raised to 18 wk. Two trials were conducted with 1,032 Lohmann Brown-Lite (**LB**) and LSL-Lite (**LW**) pullets each. A randomized complete block (trial) design was used with a 4 (perch space) × 2 (strain) factorial arrangement. Birds were raised in 16 floor pens (3 × 3 m each; 897.67 cm^2^ per bird). Wooden perches were provided from d 1, allowing 6, 9, 12, or 15 cm perch space per pullet. Data were tested for normality, and log+1 transformed if necessary. Significance was declared at *P*≤0.05. Data were collected for basic health and functioning (body weight, mortality, pullet width, keel bone damage, and tibia bone parameters), affective states (comb damage, heterophil-to-lymphocyte (**H/L**) ratios, and behavior), and natural living (perch usage and jumping success). Perch space did not affect mortality, keel bone damage, tibia breaking strength, comb damage, or H/L ratios. At 16 and 18 wk, LB body weight slightly increased while the LW body weight decreased with increasing perch space (*P*=0.05 and 0.02). At 3 wk, pullets spent a higher percentage of time wing flapping on the perch when provided 15 cm compared to 6 cm (*P*=0.04). During the scotoperiod at 18 wk, a higher percentage of birds perched with 12 and 15 cm perch space than 6 cm (*P*<0.01). Pullet width at 18 wk was 12.52 cm (sitting) and 11.66 cm (standing) for the LB and 13.85 cm (sitting) and 12.94 cm (standing) for the LW. Overall, perch space allowance had minor effects on the measured welfare indicators. Based on pullet width, a minimum of 12.5 cm for LW and 13.9 cm for LB pullets should be provided to allow all pullets to sit on the perch simultaneously.

## Introduction

Roosting on elevated structures is an anti-predator response still present in domestic table egg-producing laying hens housed in enclosed environments (Newberry et al., 2001). Laying hens are highly motivated to perch and show signs of frustration if perching is thwarted (Olsson and Keeling, 2000). In addition to fulfilling a need, the provision of perches improves bone mineralization (Hester et al., 2013), tibia bone strength (Jendral et al., 2008; Barnett et al., 2009), bone volume (Hughes et al., 1993), and decreases abdominal fat deposition (Jiang et al., 2014). Access to perches also reduces aggression (Cordiner and Savory, 2001) and fearfulness (Donaldson and O'Connell, 2012) in laying hens.

Pullet environments should be as similar as possible to their production environment to ease the transition to the laying facility, promote optimal utilization of resources, and prevent frustration and associated injurious pecking (Janczak and Riber, 2015). Therefore, the Canadian Codes of Practice for the Care and Handling of Pullets and Laying Hens (2017) requires perches for chicks reared in multi-tier systems and recommends perch provision for pullets destined for single-tier laying systems. As Canada aims to transition from conventional cages to enriched cage or non-cage housing by 2036, preparing pullets for these housing systems becomes even more important. Studies have shown that perches should be provided no later than 7 d of age (Heikkilä et al., 2006; Janczak and Riber, 2015; Skånberg et al., 2021). Early perch provision helps to develop perching behavior and pullets’ spatial cognitive abilities to navigate through more complex housing systems when transferred to the layer barn (Gunnarsson et al., 2000; Heikkilä et al., 2006). Later in the production period, pullets reared with perches had wider shanks (Yan et al., 2014), better muscle deposition (Hester et al., 2013), and bone mineralization (Enneking et al., 2012). They also showed a lower prevalence of floor eggs (Appleby et al., 1988; Gunnarsson et al., 2000) and cloacal cannibalism (Gunnarsson, 1999; Huber-Eicher and Audige, 1999).

The optimal perch space provision for laying hens ranges from 14 cm (Appleby, 1995) to 18 cm (Sandilands et al., 2009) per hen. The Canadian Codes of Practice for the Care and Handling of Pullets and Laying Hens (Canadian Codes of Practice for the Care and Handling of Pullets and Laying Hens, 2017) requires 15 cm perch space per laying hen. The current recommendations for perch space allowance in Canadian floor-raised pullets are based on laying hen data. To our knowledge, research has yet to be conducted to determine the ideal perch space allowance for pullets raised in floor pens with access to single-tier perch systems.

This study aimed to determine the impacts of perch space allowances on pullet welfare up to 18 wk of age. Welfare measures were chosen based on the three concepts of animal welfare developed by Fraser (2008), where basic health and functioning, affective states, and natural living are considered. It was hypothesized that pullets with smaller bodies and lighter weights than laying hens do not require the same perch space as adult hens. Similarly, strains with different body characteristics may differ in perch space requirements. The results will support future decisions on perch space requirements in pullets. Appropriate space allowances could reduce barn space required for perches, lowering costs per pullet and the amount of cleaning and disinfection required while ensuring pullet and hen welfare.

## Materials and methods

The University of Saskatchewan's Animal Care Committee approved the experimental protocol for this trial (AUP number: 19940248). The study was performed under the Canadian Council on Animal Care (2009) recommendations, as specified in the Guide to the Care and Use of Experimental Animals.

### Housing and management

Two trials were conducted, each housing 1,032 Lohmann Brown-Lite (**LB**) and 1,032 Lohmann Selected Leghorn-Lite (**LW**) pullets from day of hatch to 18 wk. Pullets were randomly allocated to one of 16 pens (3 × 3 m), which housed 129 pullets each (697.67 cm^2^/bird). Pens were bedded with wood shavings to a depth of 7-10 cm and contained 2 tube feeders with pans (0.36 m diameter [0−8 wk] and 0.44 m diameter [8-16 wk]) and 2 drinkers (one bucket with 5 nipples and one bell drinker). During the first wk, pullets had access to supplemental feeders and drinkers. A 4-phase commercial diet and water were provided *ad libitum*. At the hatchery, pullets were infrared beak-treated and vaccinated for Marek's Rispens, HVT-IBD, and Poulvac ST. Pullets were also vaccinated for Newcastle Bronchitis, *Salmonella enteritidis*, and *Salmonella typhimurium* at various times throughout the rearing period as per vaccine requirements. Breeder flock ages for the first trial were 31 wk (LB and LW) and 32 (LB) and 53 wk (LW) for the second trial. Room temperature at placement was 33°C during the first wk and was gradually decreased to 20°C by 5 wk, where it was maintained for the duration of the trial. Light was provided by white LED lights (3000K) set at 30 lux for the first wk and 15 lux for the remainder of the trial. An intermittent lighting program (4L:2D) resulting in 16L:8D was used for the first wk. Afterwards, a single continuous photoperiod was used starting at 14L:10D and was reduced to 8L:16D by wk 6. A 15-minute dawn-to-dusk program was used (Lohmann Breeders, 2008).

Pens were randomly assigned to 1 of 4 treatments (6, 9, 12, or 15 cm perch space), resulting in 4 pens per strain and perch treatment combination. Each pen had a single-tier perch system from d 1, allowing for 6, 9, 12, or 15 cm of perch space per pullet. Perches were constructed from 3.8 × 3.8 cm square-shaped wood boards, with edges softened (European Food Safety Authority, 2015). Perches were 243.8 cm wide by 52.1 cm high, and perch rails were spaced 25 cm apart.

### Data collection for basic health and functioning

***Body Weight.*** Pullets were weighed on a pen basis at 0, 4, 8, 12, 16, and 18 wk, and the average BW per bird was calculated (n = 16 pens per trial).

***Mortality.*** Pullets were checked twice daily throughout the trial for mortality and culls. A Humane Intervention Point Checklist was used to determine if euthanasia was necessary. All mortality were submitted to an independent diagnostic laboratory (Prairie Diagnostic Services, Saskatoon, SK, Canada) for necropsy to determine the cause of morbidity or mortality.

***Pullet Width.*** The width of 5 randomly chosen pullets per pen per trial was measured at 4, 8, 12, 16, and 18 wk. Measurements were taken at the widest part of the body (including the wings folded at the side) while standing and sitting (Riddle et al., 2018).

***Keel Bone Damage.*** At 18 wk, 30 randomly selected pullets per pen and trial were palpated for keel bone fractures and deviations using the Simplified Keel Assessment Protocol (Casey-Trott et al., 2015). The number of fractures, locations in the bone, and the presence or absence of deviations from the straight line were recorded. Two trained and blinded individuals assessed each bird and mutually agreed on the keel bone status. Three of the 30 birds per pen from the keel bone palpation were randomly chosen and euthanized via injection of T-61 (0.4 mL mebezonium iodide/tetracaine per kg of BW; Intervet Canada Corp, Kirkland, QC, Canada) into the brachial vein. The keel bones were scored for fractures and deviations (Casey-Trott et al., 2015). Like the palpation data, a mutual agreement was reached between the same 2 assessors.

***Tibia Bone Parameters.*** After keel bone removal, the right tibiae were removed, cleaned, and frozen at -20°C until further assessment of bone breaking strength (3 pullets per pen and trial). Tibia bones were thawed at 4°C for 24 h before the bone strength assessment. The weights of the bones were recorded. Tibia length and width perpendicular and parallel to the direction of the applied force were measured using a 150 mm digital caliper (Mastercraft 58-6800- 4; Mastercraft Tools, Toronto, Canada). The breaking strength of the tibia was determined using a 3-point bending test with an Instron Universal Testing Machine (Instron 3366; Instron Corp., Norwood, MA; 50 kg load cell; 30 mm/min loading rate). Bones were placed dorsal side up on supports placed 5 cm apart. The flexure load (N) was recorded, and the maximum flexure load was used as the ultimate force required to break the tibia. Post-breakage, the internal widths at the inflection point perpendicular and parallel to the direction of applied force were measured via caliper. These measurements were used to calculate the distance between the neutral axis of the bone and the extreme outer fiber, which are points along the plane of the bone (C), and the moment of inertia (Crenshaw et al., 1981). For each measurement, the absolute value was used for calculation. In addition, relative values were calculated to adjust for the birds' body weights. The flexure load was converted to kilograms (1 N = 0.010971621 kg) for further calculations:

(1) $$C=\frac{D}{2}$$

(2) $$Moment\;of\;inertia=0.0491\;\left(BD^{3}-\;bd^{3}\right)$$

(3) $$Stress\;\left(\frac{kg}{cm^{2}}\right)=\;\frac{force\;\left(kg\right)\times length\;\left(cm\right)\times C\;\left(cm\right)}{4\;\times moment\;of\;inertia\;\left(cm^{4}\right)}$$

where C = distance between the neutral axis of the bone and the extreme outer fiber, which are the points along the plane of the bone; D = outside diameter of the bone at the point of loading and parallel to the direction of the applied force; B = outside diameter of the bone at the point of loading and perpendicular to the direction of applied force; b = inside diameter of the bone at the point of loading and perpendicular to the direction of applied force; d = inside diameter of the bone at the point of loading and parallel to the direction of applied force.

### Data collection for affective states

***Comb Damage.*** At 12 and 18 wk, 30 randomly selected pullets per pen and trial were comb-scored to assess the degree of pecking damage to the comb as an indicator of aggression (adapted from Ali and Cheng, 1985), where a score of 0 indicated no sign of pecking damage, score 1 indicated a single mark of pecking damage, score 2 indicated two to three marks of pecking injuries on either side of the comb, score 3 indicated more than three marks of pecking on the comb, and Score 4 indicated severe injuries, bleeding, extensive damage to the comb. Two trained and blinded individuals assessed each bird and agreed on the comb score.

***H/L Ratios.*** Blood samples were collected from the brachial vein of 6 randomly selected pullets per pen and trial to determine the heterophil/lymphocyte (**H/L**) ratio at 12 and 18 wk. Blood smears were prepared within 30 minutes of blood collection. Blood smears were dried for 24 h, stained with PROTOCOL Hema 3 (Fisher Scientific, Ottawa, ON, Canada), and then analyzed under 100X oil magnification. Heterophils and lymphocytes were counted until a total of 100 cells were reached, after which the H/L ratio was calculated.

***Behavior on the Perch.*** Pens were video recorded for 24 h at 3, 9, 15, and 18 wk via ceiling-mounted infrared video cameras (Matrix Network Inc., Coppell, TX). From the video footage, behavior performed on the perch, perch usage, and jumping success were determined.

Behaviors performed on the perch at 3 and 15 wk were assessed using scan sampling at 20-min intervals during a 6 h photoperiod (09:00 to 16:00). Behavioral expression was classified using an ethogram (Table 1) and calculated as the percentage of time (**%t**) birds performed the behavior on the perch.

**Table 1:** Behavioral ethogram for the pullets (Adapted from Webster and Hurnik, 1990; Savory, 1995; Estevez et al., 2002; Nicol et al., 2009; De Haas et al., 2010; Ericsson et al., 2014; Hunniford and Widowski, 2018).

Behavior	Definition
Active Behaviors	
Standing	Body is upright and idle (Nicol et al., 2009)
Walking	Minimum two successive steps are taken (Webster and Hurnik, 1990)
Jumping	Both feet are off the ground (De Haas et al., 2010)
Resting Behavior	Bird is inactive and is lying or crouching with breast on the floor or the head tucked under the wing (Ericsson et al., 2014)
Comfort Behaviors	
Preening	Manipulating their own feathers with their beak while either standing or laying (Nicol et al., 2009)
Stretching	Leg or wing is extended out to the or behind the body, then returning to initial position without taking a step (Nicol et al. 2009)
Tail wagging	Tail is moved side-to-side without the rest of the body moving (Nicol et al., 2009)
Head shaking	Rapid side to side motion of the head, may be accompanied by slight raise of neck and head feathers (Nicol et al., 2009)
Head scratching	Leg is extended forward and upward to scratch the head or neck (Nicol et al., 2009)
Feather Ruffling	Shaking out the feathers of the wings and body (Ericsson et al., 2014)
Wing flapping	Extending the wings and flapping them up and down rapidly (Nicol et al., 2009)
Nutritive Behaviors	
Feeding	Head extended into the feeder while sitting or standing (Webster and Hurnik, 1990)
Drinking	Pecking at the nipple drinker (Ericsson et al., 2014)
Exploratory Behaviors	
Gentle Pecking	Pecking at other birds without causing pain/discomfort or damaging the plumage (Nicol et al., 2009)
Environmental Pecking	Pecking at the pen wall, perch, feeder tube, or drinker (not the nipples) (Nicol et al., 2009)
Aggressive Behaviors	
Aggressive pecking	Pecking directed at other birds’ heads, neck, or feet, which causes the recipient to retreat (Hunniford and Widowski, 2018)
Severe feather pecking	Pulling or tearing of a flock mates feathers leading to the feathers being broken or removed. Victim may move away or confront the pecker (Savory, 1995)
Unidentified	Behavior cannot be identified because the bird or the action of the bird cannot be seen

### Data collection for natural living

***Perch Usage.*** The 24 h video recordings were also used to assess perch usage using scan sampling at 20 min intervals during the 6 h photoperiod (09:00 to 16:00) at 3, 9, and 15 wk. In addition, a time point during the scotoperiod (00:00) at 18 wk was chosen to indicate night-time perching usage when pullet body size was largest. Perch usage was expressed as the percentage of pullets (**%p**) utilizing the perch.

***Jumping Success.*** At 3 and 18 wk, successful jumps during a 6 h photoperiod (09:00 to 16:00) were assessed via continuous behavior sampling. Successful jumps from the floor to the perch or the perch to the floor were recorded, defined by Chew et al. (2021b) as a pullet landing in its target location without incident (falling or crashing). Successful jumps were expressed as the percentage of successful jumps (**%sj**) per bird out of the total number of jumps performed.

### Statistical analyses

The experiment utilized a randomized complete block (2 trials) design in a 4 (perch space) × 2 (strain) factorial arrangement. Before analyses, data were checked for normality using the Proc Univariate in SAS 9.4 (Cary, NC). If not normally distributed, data was log+1 transformed for analysis and back-transformed for table presentation. Data were analyzed using Proc Mixed with pen as the replicate unit (8 replications for perch space; 16 replications per strain). A Tukey's range test was used to separate means. Tables include back-transformed data (means and standard errors) and replication numbers. Significance was declared when *P*≤0.05.

Due to technical issues with some of the cameras at 9 and 15 wk, replicates for behavior observations and perch usage were reduced. Therefore, data for these time points were excluded from statistical analyses, and tables with descriptive data only (means and standard deviations) were included.

## Results

### Basic health and functioning

***Body Weight.*** Providing pullets with different perch space allowances did not impact body weight at any age (Table 2). Body weight differed between strains at each age (*P*<0.01), with LW birds weighing more until 4 wk, and LB birds being heavier from 8-18 wk. An interaction between perch space and strain was noted at 16 and 18 wk (*P*=0.05 and *P*=0.02). For both interactions, the LB pullet's body weight showed a small increase, while the LW pullet's body weight decreased with increasing perch space.

**Table 2:** Body weight (kg) of Lohmann Brown-Lite (LB) and Lohmann Selected Leghorn-Lite (LW) pullets reared in floor pens with access to 6, 9, 12, or 15 cm perch space per bird at 0, 4, 8, 12, 16, and 18 wk of age.

	Perch space (P)								Strain (S)						P×S		
Age (wk)	6 (n=8)	9 (n=8)		12 (n=8)		15 (n=8)	*P*-value		LB (n=16)		LW (n=16)		*P*-value		*P*-value		SEM^1^
0	0.035	0.035		0.035		0.035	0.82		0.034^b^		0.036^a^		<0.01		0.98		0.0003
4	0.280	0.278		0.280		0.282	0.78		0.268^b^		0.292^a^		<0.01		0.75		0.0031
8	0.696	0.360		0.704		0.705	0.19		0.711^a^		0.686^b^		<0.01		0.45		0.0036
12	1.086	1.080		1.094		1.091	0.48		1.147^a^		1.028^b^		<0.01		0.11		0.0111
16	1.269	1.262		1.283		1.279	0.27		1.378^a^		1.168^b^		<0.01		0.05		0.0194
18	1.433	1.410		1.435		1.429	0.11		1.603^a^		1.251^b^		<0.01		0.02		0.0319
P×S interactions																	
	LB									LW							
Age (wk)	6 (n=4)		9 (n=4)		12 (n=4)			15 (n=4)		6 (n=4)		9 (n=4)		12 (n=4)		15 (n=4)	
16	1.36^a^		1.36^a^		1.39^a^			1.40^a^		1.18^b^		1.17^b^		1.17^b^		1.16^b^	
18	1.60^ab^		1.57^b^		1.62^ab^			1.62^a^		1.27^c^		1.25^c^		1.25^c^		1.24^c^	

1 SEM: pooled standard error of the mean

a, b Means within the same row and main effect or interaction differ significantly (*P*≤0.05)

***Mortality.*** Perch space did not affect mortality. LW pullets had lower total mortality than LB pullets (0.43% vs 2.42%; *P*<0.01), but these occurrences were unrelated to perch treatment. No mortalities or culls were due to skeletal issues.

***Body Width.*** As expected, perch treatment did not impact body width while standing or sitting (Table 3). At 8, 12, 16, and 18 wk, LB pullets were wider when standing than LW pullets (*P*<0.01). At 12, 16, and 18 wk, LB birds were wider when sitting than LW pullets (*P*<0.01).

**Table 3:** Body width measures (cm) of Lohmann Brown-Lite (LB) and Lohmann Selected Leghorn-Lite (LW) pullets reared in floor pens with access to 6, 9, 12, or 15 cm perch space per bird at 4, 8, 12, 16, and 18 wk of age while standing or sitting.

	Perch space (P)					Strain (S)			P×S	
Age (wk)	6 (n=8)	9 (n=8)	12 (n=8)	15 (n=8)	*P*-value	LB (n=16)	LW (n=16)	*P*-value	*P*-value	SEM^1^
Standing										
4	7.44	7.39	7.47	7.53	0.47	7.45	7.47	0.77	0.19	0.058
8	10.15	10.08	10.25	10.22	0.45	10.43^a^	9.92^b^	<0.01	0.88	0.130
12	11.37	11.56	11.55	11.62	0.34	12.03^a^	11.01^b^	<0.01	0.31	0.162
16	12.30	12.28	12.22	12.31	0.81	12.82^a^	11.73^b^	<0.01	0.88	0.123
18	12.27	12.26	12.31	12.35	0.87	12.94^a^	11.66^b^	<0.01	0.98	0.142
Sitting										
4	8.43	8.42	8.46	8.49	0.91	8.44	8.45	0.89	0.07	0.089
8	11.21	11.28	13.72	11.41	0.45	12.71	11.09	0.21	0.44	0.635
12	12.27	12.45	12.45	12.59	0.33	12.91^a^	11.97^b^	<0.01	0.75	0.164
16	13.17	13.28	13.26	13.22	0.82	13.79^a^	12.67^b^	<0.01	0.21	0.125
18	13.11	13.14	13.30	13.19	0.33	13.85^a^	12.52^b^	<0.01	0.58	0.134

1 SEM: pooled standard error of the mean

a, b Means within the same row and main effect differ significantly (*P*≤0.05)

***Keel Bone Damage.*** Neither perch space treatment nor strain influenced the incidence of keel deviations. No keel bone fractures were observed.

***Tibia Bone Parameters.*** Tibia parameters are provided in Table 4. The absolute outer width of the tibia was wider in birds reared with 6 cm of perch space per bird compared to 9 cm per bird (*P*=0.04). The relative thickness of the tibia was greater in birds with access to 15 cm perch space per bird compared to 6 cm (*P*=0.05). No differences were noted in the stress values based on perch space treatments. Strain impacted numerous tibia bone parameters. For absolute values, LB pullet tibiae were heavier (*P*<0.01) and longer (*P*=0.01) and had higher values pre-breakage and post-breakage for outer width (*P*<0.01), inner width (*P*<0.01), and thickness (*P*=0.01; *P*=0.03). For relative values corrected for body weight, LW pullets had longer tibias (*P*<0.01), greater pre-breakage outer width (*P*<0.01), inner width (*P*<0.01), thickness (*P*=0.03), and greater post-breakage outer and inner width (*P*<0.01; *P*=0.01). The calculation for bone strength in resistance to mechanical stress relative to bone size was also higher for LW pullet tibias than LB (*P*<0.01).

**Table 4:** Tibia bone parameters of Lohmann Brown-Lite (LB) and Lohmann Selected Leghorn-Lite (LW) pullets reared in floor pens with access to 6, 9, 12, or 15 cm perch space per bird at 16 wk of age.

	Perch space (P)					Strain (S)			P×S	
	6 (n=8)	9 (n=8)	12 (n=8)	15 (n=8)	*P-*value	LB (n=16)	LW (n=16)	*P-*value	*P* value	SEM^1^
*Absolute*										
Weight (g)	10.50	10.60	10.70	10.88	0.74	11.85^a^	9.49^b^	<0.01	0.33	0.242
Length (cm)	11.59	11.58	11.54	11.61	0.90	11.68^a^	11.48^b^	0.01	0.61	0.037
Outer width (W^2^, cm)	0.75	0.72	0.73	0.74	0.21	0.78^a^	0.69^b^	<0.01	0.79	0.010
Inner width (W^2^, cm)	0.52	0.52	0.52	0.54	0.27	0.56^a^	0.49^b^	<0.01	0.72	0.008
Thickness (W^2^, cm)	0.23	0.21	0.21	0.20	0.16	0.22^a^	0.20^b^	0.01	0.42	0.006
Outer width (N^3^, cm)	0.626^a^	0.602^b^	0.604^ab^	0.613^ab^	0.04	0.65^a^	0.57^b^	<0.01	0.20	0.0078
Inner width (N^3^, cm)	0.42	0.41	0.43	0.45	0.18	0.46^a^	0.40^b^	<0.01	0.26	0.007
Thickness (N^3^, cm)	0.20	0.19	0.17	0.17	0.08	0.19^a^	0.17^b^	0.03	0.73	0.006
Force (kg)	19.65	19.20	19.21	18.88	0.84	19.73	18.73	0.13	0.53	0.289
*Relative to BW*^4^										
Weight (g)	0.75	0.76	0.77	0.77	0.69	0.77	0.76	0.69	0.35	0.008
Length (cm)	8.40	8.35	7.46	8.41	0.95	7.59^b^	9.22^a^	<0.01	0.87	0.157
Outer width (W^2^, cm)	0.54	0.52	0.53	0.53	0.16	0.51^b^	0.55^a^	<0.01	0.85	0.005
Inner width (W^2^, cm)	0.38	0.37	0.38	0.39	0.52	0.36^b^	0.39^a^	<0.01	0.57	0.005
Thickness (W^2^, cm)	0.16^a^	0.15^ab^	0.15^ab^	0.14^b^	0.05	0.14^b^	0.16^a^	0.03	0.42	0.003
Outer width (N^3^, cm)	0.45	0.43	0.44	0.44	0.49	0.42^b^	0.46^a^	<0.01	0.95	0.005
Inner width (N^3^, cm)	0.30	0.30	0.32	0.32	0.28	0.30^b^	0.32^a^	0.01	0.70	0.005
Thickness (N^3^, cm)	0.15	0.14	0.12	0.12	0.06	0.13	0.14	0.15	0.62	0.004
Stress (kg/cm^2^)	1105.76	1246.53	1301.59	1256.56	0.10	1019.43^b^	1435.79^a^	<0.01	0.79	46.888

1 SEM: pooled standard error of the mean

2 W: wide. The diameters are perpendicular to the direction of the applied force

3 N: narrow. The diameters are parallel to the direction of the applied force

4 BW: body weight

a, b Means within the same row and main effect differ significantly (*P*≤0.05)

### Affective states

***Comb Damage.*** Comb damage was unaffected by perch treatment at 12 and 18 wk. At 12 wk, LW birds (0.52 ± 0.043) had poorer comb sores compared to LB pullets (0.27 ± 0.044; *P*<0.01).

***H/L Ratios.*** H/L ratios were unaffected by perch space allowance. At both 12 (LB: 0.33 ± 0.013; LW: 0.16 ± 0.012) and 18 wk (LB: 0.58 ± 0.026; LW: 0.47 ± 0.030), LB pullets had higher H/L ratios compared to LW birds (*P*<0.01).

***Behavior on the Perch.***Table 5 presents the %t birds performed behaviors on the perch at 3 wk of age, where birds spent a higher %t wing flapping in the 15 cm treatment compared to 6 cm (*P*=0.01). LW pullets performed a higher %t of standing (*P*=0.01), jumping (*P*=0.03), preening (*P*=0.04), active (*P*=0.02), and comfort (*P*=0.02) behaviors compared to LB pullets. No incidences of tail wagging, feather-ruffling, feeding, or drinking, were observed at 3 wk of age.

**Table 5:** Behavior (% of time) on the perch of Lohmann Brown-Lite (LB) and Lohmann Selected Leghorn-Lite (LW) pullets reared in floor pens with access to 6, 9, 12, or 15 cm perch space per bird during the photoperiod (09:00-16:00) at 3 wk of age.

	Perch space (P)					Strain (S)			P×S	
	6 (n=8)	9 (n=8)	12 (n=8)	15 (n=8)	*P*-value	LB (n=16)	LW (n=16)	*P*-value	*P*-value	SEM^1^
Active^2^	64.40	62.92	68.38	64.74	0.76	63.41^b^	66.81^a^	0.02	0.92	3.789
Standing	57.53	50.07	60.21	54.90	0.49	54.98^b^	56.37^a^	0.01	0.77	3.439
Walking	6.06	11.91	7.68	8.00	0.59	8.00	8.82	0.06	0.16	1.619
Jumping	0.82	0.94	0.49	1.85	0.25	0.43^b^	1.62^a^	0.03	0.86	0.286
Resting	10.95	14.03	11.37	13.40	0.57	15.44	9.44	0.54	0.91	2.311
Comfort^3^	22.04	18.76	17.32	18.46	0.95	17.27^b^	21.02^a^	0.02	0.38	2.175
Preening	21.80	18.09	16.68	15.86	0.95	16.48^b^	19.74^a^	0.04	0.41	2.219
Wing flapping	0.25^b^	0.45^ab^	0.59^ab^	2.60^a^	0.01	0.69	1.25	0.07	0.66	0.282
Head scratching	0.00	0.08	0.00	0.00	0.41	0.00	0.04	0.33	0.41	0.019
Head shake	0.00	0.00	0.05	0.00	0.41	0.02	0.00	0.33	0.41	0.011
Stretching	0.00	0.15	0.00	0.00	0.41	0.07	0.00	0.33	0.41	0.037
Exploratory^4^	0.98	3.83	1.27	0.32	0.65	2.01	1.19	0.62	0.38	0.803
Environmental pecking	0.84	3.56	1.23	0.17	0.60	1.99	0.91	0.99	0.37	0.802
Gentle feather pecking	0.14	0.27	0.05	0.15	0.85	0.02	0.28	0.09	0.52	0.068
Aggressive	0.00	0.10	0.00	0.02	0.54	0.00	0.06	0.19	0.54	0.026
Severe feather pecking	0.00	0.10	0.00	0.02	0.54	0.00	0.06	0.19	0.54	0.026
Unidentified	1.62	0.36	1.67	3.05	0.49	1.88	1.47	0.48	0.56	0.649

1 SEM: pooled standard error of the mean

2 Sum of standing, walking, and jumping behavior

3 Sum of preening, wing flapping, head scratching, head shaking, and stretching behavior

4 Sum of environmental pecking and gentle feather pecking behavior

a, b Means within the same row and main effect differ significantly (*P*≤0.05)

Due to camera issues, replications for the 15 wk behavior observations were too low to conduct statistical analyses. Therefore, descriptive data (means and standard deviations) are presented in Table 6.

**Table 6:** Descriptive results for the behavior (% of the time) of Lohmann Brown-Lite (LB) and Lohmann Selected Leghorn-Lite (LW) pullets reared in floor pens with access to 6, 9, 12, or 15 cm perch space per bird during the photoperiod (09:00-16:00) at 15 wk of age.

	Perch space								Strain			
	6 (n=5)		9 (n=5)		12 (n=4)		15 (n=5)		LB (n=7)		LW (n=12)	
	Mean	SD^1^	Mean	SD^1^	Mean	SD^1^	Mean	SD^1^	Mean	SD^1^	Mean	SD^1^
Active^2^	38.86	13.814	43.74	13.581	44.13	9.212	43.53	5.156	53.31	5.636	36.17	5.991
Standing	38.14	12.727	43.06	13.488	43.46	8.688	42.68	4.735	52.11	5.569	35.71	6.075
Walking	0.56	0.449	0.56	0.317	0.53	0.631	0.74	0.606	1.06	0.464	0.33	0.174
Jumping	0.16	0.117	0.12	0.117	0.14	0.111	0.12	0.078	0.13	0.097	0.13	0.104
Resting	19.04	8.045	12.11	6.053	15.39	3.678	15.52	4.067	9.95	3.879	18.77	4.473
Comfort^3^	29.61	4.731	26.39	5.536	29.13	2.803	29.65	3.984	25.07	3.770	30.78	3.103
Preening	28.85	4.841	25.64	5.318	28.18	2.866	28.97	4.109	24.25	3.881	30.02	2.975
Wing flapping	0.18	0.137	0.36	0.283	0.27	0.219	0.29	0.261	0.40	0.315	0.21	0.116
Head scratching	0.09	0.208	0.12	0.137	0.12	0.094	0.15	0.173	0.12	0.165	0.12	0.148
Tail wagging	0.20	0.237	0.10	0.158	0.22	0.095	0.13	0.127	0.17	0.229	0.15	0.113
Feather ruffle	0.02	0.053	0.03	0.062	0.02	0.049	0.00	0.000	0.00	0.000	0.03	0.054
Head shake	0.00	0.000	0.00	0.000	0.05	0.056	0.01	0.031	0.00	0.000	0.02	0.040
Stretching	0.27	0.068	0.13	0.131	0.28	0.182	0.10	0.123	0.13	0.185	0.22	0.107
Exploratory^4^	0.92	0.068	1.49	0.901	1.55	0.308	1.37	0.371	1.31	0.773	1.33	0.393
Environmental pecking	0.14	0.144	0.70	0.604	0.59	0.101	0.45	0.172	0.63	0.517	0.36	0.238
Gentle feather pecking	0.78	0.151	0.80	0.436	0.96	0.302	0.91	0.305	0.68	0.308	0.96	0.222
Nutritive^5^	0.15	0.346	0.47	0.502	0.03	0.067	0.31	0.657	0.54	0.609	0.08	0.222
Feeding	0.15	0.346	0.47	0.502	0.02	0.035	0.28	0.579	0.52	0.566	0.08	0.221
Drinking	0.00	0.000	0.00	0.000	0.02	0.032	0.03	0.078	0.02	0.066	0.01	0.019
Aggressive	0.00	0.000	0.02	0.038	0.00	0.000	0.00	0.000	0.00	0.000	0.01	0.025
Aggressive pecking	0.00	0.000	0.02	0.038	0.00	0.000	0.00	0.000	0.00	0.000	0.01	0.025
Unidentified	11.42	6.317	15.79	9.868	9.76	4.867	9.62	3.127	9.83	6.800	12.87	12.866

1 SD: standard deviation

2 Sum of standing, walking, and jumping behavior

3 Sum of preening, wing flapping, head scratching, head shaking, and stretching behavior

4 Sum of environmental pecking and gentle feather pecking behavior

5 Sum of feeding and drinking behavior

### Natural living

***Perch Usage.***Table 7 shows the %p using the perch during the photoperiod at 3 wk and the scotoperiod at 18 wk. Perch space did not affect the %p on the perch at 3 wk, but LW pullets perched more than LB pullets (*P*<0.01). At 18 wk, the %p on the perch during the scotoperiod was higher in the 12 and 15 cm perch space per bird treatments compared to the 6 cm perch space per bird treatment (*P*<0.01), and more LW birds were observed perching compared to LB pullets (*P*<0.01).

**Table 7:** Perch usage (% of the pullets) of Lohmann Brown-Lite (LB) and Lohmann Selected Leghorn-Lite (LW) pullets reared in floor pens with access to 6, 9, 12, or 15 cm perch space per bird at 3 weeks during the photoperiod (09:00-16:00) and 18 wk during the scotoperiod (00:00).

	Perch space (P)					Strain (S)			P×S	
Age (wk)	6 (n=8)	9 (n=8)	12 (n=8)	15 (n=8)	*P*-value	LB (n=16)	LW (n=16)	*P*-value	*P*-value	SEM^1^
Photoperiod										
3	0.8	1.7	1.7	1.9	0.29	0.7^b^	2.3^a^	<0.01	0.70	0.25
Scotoperiod										
18	33^b^	49^ab^	51^a^	63^a^	<0.01	30^b^	68^a^	<0.01	0.77	4.5

1 SEM: pooled standard error of the mean

a, b Means within the same row and main effect differ significantly (*P*≤0.05)

Data for perch usage during 9 and 15 wk were excluded from statistical analyses due to camera issues and the resulting low replications. Table 8 provides the descriptive results for the %p perching during the photoperiod at 9 and 15 wk of age.

**Table 8:** Descriptive results of perch usage (% of the pullets) of Lohmann Brown-Lite (LB) and LSL-Lite (LW) pullets reared in floor pens with access to 6, 9, 12, or 15 cm perch space per bird at 9 and 15 weeks during the photoperiod (09:00-16:00).

	Perch space								Strain			
Age (wk)	6		9		12		15		LB		LW	
	Mean	SD^1^	Mean	SD^1^	Mean	SD^1^	Mean	SD^1^	Mean	SD^1^	Mean	SD^1^
9	(n=6)		(n=7)		(n=7)		(n=7)		(n=14)		(n=13)	
	14	5.9	19	7.3	23	5.7	20	7.5	16	5.8	22	6.8
15	(n=5)		(n=5)		(n=4)		(n=5)		(n=7)		(n=12)	
	22	4.4	25	10.9	36	8.9	37	13.9	19	3.6	36	9.8

1 SD: standard deviation.

***Jumping Success.*** The %sj per pullet from the floor to the perch or the perch to the floor was unaffected by perch space at 3 and 18 wk. At 3 wk, when jumping from the floor to the perch, the LB strain had a higher %sj per pullet (99.95 vs 99.57%) compared to LW pullets (*P*=0.01). No strain differences existed at 18 wk.

## Discussion

### Basic health and functioning

Perch space allowance did not impact body weight at any measured age. This agrees with Campbell et al. (2020), who compared perch access to no perch access and found no differences in body weight at 5, 8, 12, and 16 wk. However, Enneking et al. (2012) reported increased body weight at 12 wk when pullets were given access to a perch (10 cm/bird). LW pullets were heavier early in life (0 and 4 wk), whereas LB pullets were heavier from 8 wk onward. LB pullets have a larger body size than LW pullets, as evidenced by the current study's body weight and width data. The LB strain was significantly heavier than LW, which aligns with previous research that reports brown-feathered laying hens were heavier than white-feathered birds (Riczu et al., 2004; Chew et al., 2021a). Interactions observed between body weight and perch space at 16 and 18 wk suggest that LW body weight slightly decreases when birds are provided with more perch space, while LB body weight slightly increases. It should be noted that the differences in body weight for each interaction were not substantial for either the LB (16 wk=40 g; 18 wk=50 g) or LW strains (16 wk=20 g; 18 wk=30 g).

Mortality was unaffected by perch space, which agrees with Hester et al. (2013a), who compared birds with no perch access, access during the pullet or laying phase, and access throughout the bird's life cycle. LW pullets had increased total mortality and a higher percentage of mortalities due to infectious causes. It is possible that differences in parent flock age can explain this (Yerpes et al., 2020). For trial 1, the parent flock ages for both the LB and LW birds was 31 wk. For trial 2, the parent flock age for the LB pullets was 32 wk, whereas for the LW pullets it was 53 wk.

The average pullet width at 18 wk for both strains was 12.30 cm when standing and 13.19 cm when sitting, which are both lower than the perch space recommendation of 15 cm given by the Canadian Codes of Practice for the Care and Handling of Pullets and Laying Hens (2017). As expected, the LB pullets were wider than the LW pullets at most ages, with 18 wk averages of 12.94 vs 11.66 cm (standing) and 13.85 vs 12.52 cm (sitting). Giersberg et al. (2017) also reported that brown feathered pullets (Lohmann Brown and Lohmann Tradition) were wider than white feathered pullets (Lohmann LSL-Lite) when standing at 8 and 19 wk and while sitting at 19 wk. This strain effect was also noted in laying hens (Riddle et al., 2018). Therefore, strain differences must be accounted for when determining perch space allowance.

In the current study, neither perch space nor strain impacted keel bone deviations at 18 wk. This aligns with Hester et al. (2013), who reported no impact of perch access or no access in the pullet phase on keel bone fractures or keel scores at 71 wk. Keel bone growth and ossification are slow processes that continue into the laying phase until approximately 28-40 wk of age; therefore, the caudal portion of the keel is still cartilaginous during the pullet phase (Buckner et al., 1948). This may account for the lack of deviation during the current study.

Perch space had a minor effect on tibia parameters in the current study. These differences were numerically small, likely having no biological relevance, and no impact on force or stress was noted because of the differences. No literature assessing the impact of perch space on pullet bone health could be found, and the literature regarding hens is not in agreement. Hens who were given access to perches as pullets had wider shanks than those without access (Yan et al., 2014). Hester et al. (2013) found that access to perches during the pullet phase increased muscle deposition in 71 wk hens. Enneking et al. (2012) reported that access to perches increased the bone mineral content of the tibia and the left leg muscle weight at 12 wk, suggesting that perch access improved leg health. In the current study, the white feathered strain had a higher resistance to bone stress, which agrees with Chew et al. (2021a).

### Affective states

Comb damage was evaluated as an indicator of aggression, and no differences were noted between perch treatments. No previous studies assessing the impact of perch space on pullet aggression have been published to our knowledge. However, giving laying hens access to 15 cm perch space per bird reduced aggression (Cordiner and Savory, 2001; Donaldson and O'Connell, 2012). It was suggested that subordinate birds used the perches to avoid dominant birds during the photoperiod, which may have accounted for the reduced aggression (Cordiner and Savory, 2001). However, Guhl (1956) described that dominant hens have the privilege of perching while subordinates are relegated to the floor area.

H/L ratio is a measure of environmental stress (Gross and Siegel, 1983) and was unaffected by perch space allowance at 12 and 18 wk in the current study, which agrees with Campbell et al. (2020), who compared pullets reared under no enrichment to those with perch access at 15 and 16 wk. Yan et al. (2013) also reported no differences in multiple stress parameters (epinephrine, norepinephrine, dopamine, corticosterone, serotonin) in white leghorn pullets with or without perch access at 4, 6, and 12 wk. The comb damage and H/L ratio results from this project suggest that perch space did not influence aggression.

The past literature on perching in pullets primarily focused on perch access or no access. Therefore, no previous research assessed the behavioral repertoire of pullets on the perch. In the current study, birds spent a higher %t wing flapping on the perch in the 15 cm treatment compared to 6 cm at 3 wk of age, but this may be related to the number of rails in the higher perch space treatment pens. Although not significant, the %t spent jumping also followed this pattern. Therefore, the higher %t wing flapping may have been due to post-jump wing flapping.

### Natural living

In the current study, no difference in the %p on the perch during the photoperiod was observed at 3 wk. At 15 wk, the %p on the perch during the photoperiod ranged from 22-37%. Previous work in hens has identified a wide range of perching during the photoperiod from 10-41% (Appleby et al., 1992; Newberry et al., 2001; Liu et al., 2018). Keeling et al. (2017) reported that perching during the photoperiod was the least synchronized behavior compared to feeding, drinking, and preening. Liu et al. (2018) noted that attaining values for the percentage of birds using the perch from manual observations (live or video) using scan sampling may not represent the actual perch usage, considering the wide variation in perching during the photoperiod. Appleby et al. (1995) offered ISA Brown hens (18-72 wk) perch space of 12, 13, 14, or 15 cm/bird and reported no difference in perching during the photoperiod.

Many studies reported an increase in perching during the scotoperiod compared to the photoperiod (Liu et al., 2018; Giersberg et al., 2019). Hens are highly motivated to perch at night (Olsson and Keeling, 2000). When provided 12 cm perch space per hen, it has been reported that up to 100% of birds utilized the perch during the scotoperiod (Tauson, 1984). The percentage of hens perching during the scotoperiod increased from 72-78% to 99% when the perch space per bird was increased from 15 to 22.5 cm (Duncan et al., 1992). Additionally, Cook et al. (2011) found that more birds perched when provided with 17, 19, and 25.8 cm/bird compared to 15 cm/bird of perch space. Wall and Tauson (2007) reported that a higher percentage of hens provided with 12 cm/bird perch space perched during the scotoperiod compared to 15 cm/bird. However, the authors concluded that this might have been due to differences in perch arrangements. In the current study, the %p perching during the scotoperiod at wk 18 increased as perch space increased. However, the range of birds on the perch was wider than in previous studies (11-95%; data not shown). The %p that were able to utilize the perch in each treatment was 46% (6 cm/bird), 69% (9 cm/bird), 90% (12 cm/bird), and 114% (15 cm/bird). Therefore, perch space was available in all treatments for more pullets to perch. The previous studies assessed perch usage in hens and not pullets, so pullets may have a lower motivation to perch than hens. Additionally, many previous studies were conducted in cage systems, whereas the current study assessed floor-reared pullets with access to single-tier perch systems. Furthermore, high individual variation for time hens spent perching has been reported (Duncan et al., 1992; Lambe and Scott, 1998), and some hens do not use the perch, preferring to roost on the floor (Appleby et al., 1992).

Strain differences were observed in the current study, where a higher %p was observed perching for the LW strain than LB during the photoperiod at 3 wk and scotoperiod at 18 wk (*P*<0.01). These findings align with the literature for hens, reporting that White leghorn pullets perched more than Brown leghorns during the photoperiod at 15 wk (Faure and Bryan Jones, 1982), and a higher percentage of Lohmann Selected Leghorn (85.9%) were observed on the perch compared to Lohman Brown hens (81.1%) during the scotoperiod from 20 to 80 wk of age (Wall and Tauson, 2007).

Perch space did not affect the percentage of successful jumps in the current study. Literature showed that brown-feathered hens (Lohmann Brown and Lohmann Traditional) had a lower proportion of safe landings compared to white-feathered hens (LSL-Lite) (Scholz et al., 2014). Chew et al. (2021b) also found that LW pullets had a higher average number of successful jumps than LB pullets when jumping from the floor to the perch or vice versa. This disagrees with the current study, where at 3 wk, LB pullets were more successful when jumping from floor to perch. However, the differences were very small (99.95% vs. 99.57%), and no strain differences existed at 18 wk.

### Implications of findings for practice

Overall, the impact of perch space on measured welfare indicators was found to be minor, complicating the process of drawing clear conclusions. Appleby (2004) argued that perching behavior in laying hens is often synchronous, with the necessary space determined by body width.

Our study revealed that the %t pullets spent perching during the scotoperiod increased with greater perch space provision at 18 wk. Nevertheless, this percentage remained highly variable, ranging from 11% to 95%. While hens tend to seek closeness or contact when roosting, this behavior alone does not justify providing fewer perch opportunities. Indeed, observations from Lill (1968) showed that most hens did not settle in direct contact with each other when given sufficient space.

Recommendations for perch space allowance in floor pens with single-tier perching systems will depend on producers' priorities regarding Fraser's three conceptions of animal welfare (Fraser, 2008). Considering the goal of allowing all pullets to perch simultaneously, a minimum of 12.5 cm for LW pullets and 13.9 cm for LB pullets would be necessary, accounting for strain differences and whether the bird is standing or sitting. These findings provide valuable insights for optimizing perch space allocation to promote pullet welfare during rearing.

## Disclosures

The authors declare no conflict of interest.
